# What Are the Current Audiological Practices for Ototoxicity Assessment and Management in the South African Healthcare Context?

**DOI:** 10.3390/ijerph17072613

**Published:** 2020-04-10

**Authors:** Katijah Khoza-Shangase, Nothando Masondo

**Affiliations:** Department of Speech Pathology and Audiology, School of Human and Community Development, University of the Witwatersrand, Johannesburg 2050, South Africa; 1438684@students.wits.ac.za

**Keywords:** assessment, current, guidelines, hearing loss, high-frequency: management, practices, ototoxicity, medication, sensorineural, South Africa

## Abstract

The study was an initial exploration of the current ototoxicity assessment and management practices by audiologists in South Africa. An exploratory survey research methodology through a cross-sectional research design was adopted where audiologists were recruited from professional associations’ databases in South Africa, using specific inclusion criteria. The study made use of an 18-item web-based survey guided by the Health Professions Council of South Africa (HPCSA) (2018) guidelines which were developed from reviewing international guidelines such as the American Speech-Language-Hearing Association (ASHA,1994) and the American Academy of Audiology (AAA, 2009). The study surveyed 31 audiologists from across the country. Data were analyzed through descriptive statistics. Findings implied significant gaps between knowledge and translation of this knowledge into practice. Over two thirds of the participants engage with ototoxicity monitoring and management, but the practices adopted by them do not align with international standards nor with the national HPCSA guidelines on assessment and management of patients on ototoxic medications. Most participants do not conduct baseline assessments, and the frequency of monitoring is irregular and reduced from the recommended; thus influencing ability for early detection and intervention of ototoxicity within this context. Non-standard assessment battery is used for assessment and monitoring, raising questions about the reliability and validity of the data used to make preventive treatment decisions. Lack of collaborative work between audiologists and the rest of the clinical team involved in the treatment of patients on ototoxic medications was found to be an important contributing factor to the less than optimal ototoxicity management practices. Of factors potentially influencing adherence to guidelines, the institution of employment, specifically employment in a tuberculosis hospital, seemed to have a positive influence, possibly due to the focused nature of the audiologists’ scope of practice there as well as availability of resources. The level of education appeared to have no influence. Current findings provide contextually relevant evidence on ototoxicity assessment and management within this context. They raise important implications for guidelines adherence and translating knowledge, policies and guidelines into practice, clinical assessment and management protocols followed, appropriate resource allocation per programme, as well as strategic planning for national ototoxicity assessment and management programmes in context. The findings also raise important implications for low- and middle-income countries, in terms of adopting international guidelines without considering context.

## 1. Introduction

Ototoxicity is a reaction to the pharmaceutical drugs used to treat communicable diseases that affects the cochlea or auditory nerve, which is characterized by vestibular or cochlea dysfunction [1]. The prevalence of medication-induced hearing loss has tripled over the years and this is attributed to increased usage of ototoxic-medications. Ototoxic medications play an important role in modern medicine; however, they have the capacity to cause significant morbidity [2]. This is particularly problematic because one of the key challenges encountered in ototoxicity assessment and management is a delay in diagnosing ototoxicity; with diagnosis occurring too late when the hearing loss has become severe and affects the speech frequencies [1].

There are over 600 categories of medications that may cause ototoxicity, and this includes aminoglycosides antibiotics, platinum-based chemotherapeutic agents, loop diuretics, macrolide antibiotics and antimalarials [1]. Ototoxicity affects people of all ages, but it is problematic to document the global scale of its occurrence due to the varied research methodologies followed, especially in low and middle income (LAMI) countries; and the diverse criteria used to define ototoxicity. Published evidence has looked at defining ototoxicity and developing early detection and monitoring guidelines to allow for considerations of treatment alterations in order to lessen or even prevent irreversible drug-induced hearing loss. Although many of these guidelines exist, their practicality, especially in LAMI countries, remains questionable, due to several factors.

In South Africa, a LAMI country with limited resources for basic healthcare and basic services, there has been no standardized practice for ototoxicity detection and monitoring. Until as recently as 2018, audiologists were adapting protocols from high income countries, such as the United States of America, to implement within the South African context, and this has often been with no or limited positive outcomes [3]. This practice led to inconsistent service delivery confusion in terms of defining ototoxicity as well as inconsistent treatment options available to patients. In 2018, the Health Professions Council of South Africa’s Speech Language and Hearing Professions Board (HPCSA) [4] issued national guidelines for ototoxicity assessment and management. Current authors, however, believe that the efficient implementation of these guidelines requires definition of the current practices around ototoxicity assessment and management for this population, and establishing how the current practice compares to the recommended guidelines. Research into this area will identify the gaps, define areas for improvement, and highlight areas requiring increased awareness and training to enhance both quantity and quality of lives of patients on ototoxic medications.

Landier [5] approximated the occurrence of ototoxicity in patients on ototoxic-medications to be between 4% to 90% people in the United States. These medications are used to treat cancer, human immunodeficiency virus (HIV), tuberculosis (TB) and other communicable diseases. A 2016 global report estimated that 10.4 million people were diagnosed with TB in 2016. Of these people, 90% were adults and 74% were residing in African countries [6]. Furthermore, the World Health Organization (WHO) [7] estimates that there are 33 million persons living with HIV in Eastern and Southern Africa, and reports a steady increase in persons living with cancer.

Despite efforts to control the amount of new cases, there was an increase in new TB cases that are thought to have occurred in 2012 [8,9]. Additionally, 65% of the patients had hypertension, 22% had multi-morbidity, and a high number of young people were on antiretroviral therapy [10]. HIV, TB, and cancer remain three of South Africa’s greatest burdens of disease. The dramatic increase in the number of people with communicable diseases has led to an increase in the usage of ototoxic-medications thus a heightened need for efficient ototoxicity monitoring and management protocols within this context.

A 2010 South African study by Khoza-Shangase [11] explored the need for ototoxicity monitoring in patients with HIV/AIDS. The study highlighted that the lack of informational counselling, resources, staff, and economic challenges affect the adequate services dedicated to ototoxicity monitoring. Therefore, very few of the patients on ototoxic-medications receive audiological monitoring. The study raised the need for ototoxicity assessment and management within this LAMI country population [11].

Although ototoxicity is argued to be non-life-threatening, it affects communication and health-related quality of life indicators with significant educational, occupational, and social consequences [3,11]. In children, ototoxicity can impact language, speech, social and cognitive development which could negatively impact on school performance and psychosocial functioning [4]. The aim of ototoxicity rehabilitation should be early identification, continual monitoring, minimizing or preventing hearing impairment and planning of appropriate early rehabilitative measures [11].

The biggest challenge encountered in ototoxicity assessment and management, is a delay in diagnosing ototoxicity due to its varied and highly inconsistent presentation [1,3,4]. Other factors like age, medical conditions and cognitive levels, and culturally influenced health-seeking behaviors may delay the early detection [1,2,8]. According to the HPCSA guidelines [4], ototoxicity assessment should begin with informational counselling which entails making patients aware of significant symptoms which need to be reported to attending audiologists and physicians. Ototoxicity often progresses undetected until significant deterioration in frequencies for speech is noted, therefore ototoxicity is better diagnosed clinically by comparing audiometric results done before and after the administration of drugs.

International ototoxicity monitoring guidelines have been developed for high-income countries These guidelines set the stage for what should be done within these countries, and the current authors argue that such protocols are suitable for contexts that are well-resourced with equipment, personnel, collaboration amongst team members, as well as efficient informational control and efficient healthcare services. In LAMI countries, the implementation of these protocols has challenges, unless contextual realities are confronted and taken cognizance of. This implementation will need to be guided by support from contextualized research evidence.

The HPCSA [4] guidelines, which are comparable with other relevant guidelines outside South Africa, e.g., ASHA and AAA guidelines, recommend that the assessment and management of ototoxicity begins with pre-treatment counselling concerning the risk of ototoxic effects from the medication. These guidelines also recommend that the patient should receive baseline measures where their current hearing thresholds are obtained. These baseline findings are to be used later for comparison with the during and post-treatment hearing thresholds. The baseline measures should include case history, otoscopy, pure-tone audiometry (frequencies between 250Hz−8000Hz in addition to 9000Hz and 125000Hz and beyond) and diagnostic distortion product otoacoustic emissions (DPOAEs) [4]. The guidelines further recommend that after the patient’s pre-treatment thresholds have been obtained, the patient should receive bi-weekly audiological evaluations. These evaluations should comprise of bilateral otoscopic examinations, bilateral pure tones (including supra-threshold testing) and bilateral diagnostic DPOAEs. Should the patient’s hearing thresholds be worse, relative to the baseline thresholds, a comprehensive audiological assessment should be carried out within the next 24 hours or before the next administration of medications [4]. The determination criteria (grading) for changes in the patient’s hearing, due to treatment, should be decided upon beforehand. Once the grading has been decided upon, the severity of the shift has to be graded according to the adverse event scale that is specific to hearing [4]. Moreover, the HPCSA guidelines state that follow-up evaluations should be repeated once a month if a shift was detected, until the hearing thresholds stabilize, and no further change is detected.

According to the HPCSA guidelines, the medical management and aural rehabilitation of the patient includes a multidisciplinary team [4] that has a key objective of adopting a comprehensive management plan for the patient, which includes developing non-ototoxic agents as one of its goals [4]. For the HPCSA [4] guidelines to be implemented efficiently, the current authors believe it is important to establish the current practice and identify gaps in this practice in order to be able to implement contextually responsive interventions for programme practicability and feasibility; hence this study.

## 2. Materials and Methods

### 2.1. Aim and Objectives

The aim of the study was to investigate the current ototoxicity assessment and management practices by South African audiologists. The specific objectives included exploring practices around the performance of baseline measures, frequency of assessments during monitoring, types of assessment measures used in the assessment batteries, criteria used to establish ototoxicity and the grading systems adopted, ototoxicity management followed with patients diagnosed with ototoxicity, as well as practices around collaborative work within this population.

### 2.2. Research Design

The study made use of a quantitative survey research methodology with a cross-sectional research design [12]. A survey methodology was appropriate for this research study because it provided the researchers with access to participants from different areas of South Africa, therefore, allowing for ease of generalizability.

### 2.3. Participant Description

#### 2.3.1. Sampling Strategy

A non-probability purposive sampling strategy was used to recruit participants where specific inclusion/ exclusion criteria were adopted. The South African Speech Language Hearing Association (SASLHA) as well as the South African Association of Audiology (SAAA) were contacted to gain access to their members’ contact details nationally in order to distribute the survey to audiologists registered with the associations who are also all registered with the Health Professions Council of South Africa (HPCSA) from across the country. Furthermore, assistance was obtained from various Heads of audiology departments at public and private hospitals to send out the survey to various members of their staff.

#### 2.3.2. Participants’ Inclusion/Exclusion Criteria

The following inclusion and exclusion criteria were used to select participants:

#### 2.3.3. Inclusion Criteria

All participants were required to be registered with the HPCSA as an audiologist or speech therapist and audiologist.

The participants were to be graduates from a South African University that offers the audiology degree.

All participants were to have been practicing audiology in either private and/or public setting.

#### 2.3.4. Exclusion Criteria

Participants who only practice in pediatric audiology were excluded from the study.

Participants who were speech therapists and audiologists who were only practicing in speech-language therapy were excluded from the research study.

#### 2.3.5. Sample Size

The current research aimed to recruit a minimum of 50 participants but only succeeded in obtaining 31 participants. The numbers of speech-language and hearing professionals registered with the Health Professions Council of South Africa (HPCSA) by January of 2020; for the entire South African population indicates a significant demand: capacity challenge; with Gauteng, one of the well-resourced provinces in the country, reflecting 233 audiologists registered for the whole province. These audiologists are spread between both the public and private sectors. As this was not an experimental design, power analysis of the sample was not conducted; however, consultation with a statistician led to a conclusion that based on the population size of 788 audiologists registered with the HPCSA in the country, with less than a quarter of them working in ototoxicity, the current sample size was deemed sufficient as it represented more than 10% of the population surveyed.

### 2.4. Data Collection

#### 2.4.1. Data Collection Tools

Online Survey: The researcher believed that an online survey that provided them access to a larger population was appropriate for this study [13]. Thus, the study made use of an online survey containing closed ended questions. The study used an 18-item survey developed by the researchers, guided by the HPCSA guidelines [4] for ototoxicity monitoring and management. The questionnaire addressed aspects including demographic information, ototoxicity assessment and management practices, the referral pathways, availability of resources, as well as facilitators and barriers to ototoxicity assessment and management. Furthermore, the possible influence of language and culture in the process, and the informational management and the quality measures available for the South African context were included in the survey (Appendix A—reflects the part of the questionnaire specific to this paper).

Ethical considerations: Prior to commencement of the study, ethical clearance was obtained from the University’s Human Research Ethics Committee (Non-Medical) (Protocol Number: STA_2019_04). The study adhered to the Helsinki Declaration of 1975, as revised in 2008 as far as ethical considerations were concerned.

#### 2.4.2. Data Collection Procedures

Once ethical clearance was obtained, the researcher contacted SASLHA and SAAA so that the online survey could be sent out to practitioners who are registered with the associations around the country. The survey was sent out through the use of Google drive. Once all surveys were completed, the researcher commenced with data analysis.

### 2.5. Data Analysis

#### 2.5.1. Methods of Analysis and Presentation of Data

Data were analyzed using descriptive statistics. Descriptive statistics gave numerical and graphical methods to summarize the data in a clear and understandable way [14]. This information is represented in the form of modes, medians and averages.

#### 2.5.2. Reliability and Validity

The study ensured reliability by administering the same questionnaire in the survey for all the participants. In addition to this, efforts to increase likelihood of success of the main study were implemented by conducting a pilot study [15]. The pilot study aimed to test the self-developed survey prior to the main study to determine its feasibility, adequacy, reliability and validity [15]. This survey had been adapted from similar surveys in the area of current practices in South Africa [16,17].

## 3. Results and Discussion

### 3.1. Description of the Sample

**Figure 1:**
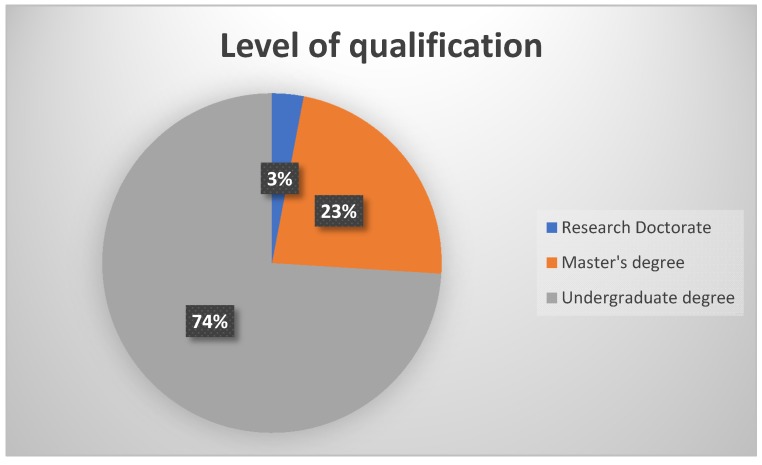
Participants’ level of highest qualification.

A total of 31 participants responded to the survey. As depicted in Figure 1, 74% (n = 23) of the participants had an undergraduate degree in audiology or speech therapy and audiology, with the rest (n = 8) having obtained a postgraduate degree in audiology (either a Master’s degree or a Research Doctorate).

This demographic profile is representative of the South African audiology community which has more practitioners with an undergraduate qualification than post-graduate qualification. In South Africa, a postgraduate qualification is not a requirement for registration for clinical practice. Of the total sample, 71% (n = 22) of the participants reported to work in the public health sector, with a third 29% (n = 9) working in the private health sector. The fact that a majority of the sample was in the public sector is a positive finding for the current study as this is the sector that provides clinical services to at least 80% of the South African population, Therefore, evidence from this group has greater applicability in terms of guidelines implementation to the majority of the country’s citizens. It is acknowledged that this sector placement profile has no relationship to the actual placement of audiologists in the South African context. It is merely a feature of who completed the survey. Anecdotally from National Forums, as these data are not collected by the HPCSA, a large majority of audiologists in South Africa are in the private sector, with documented capacity versus demand challenges in the public sector.

### 3.2. Performance of Baseline Measures

**Figure 2:**
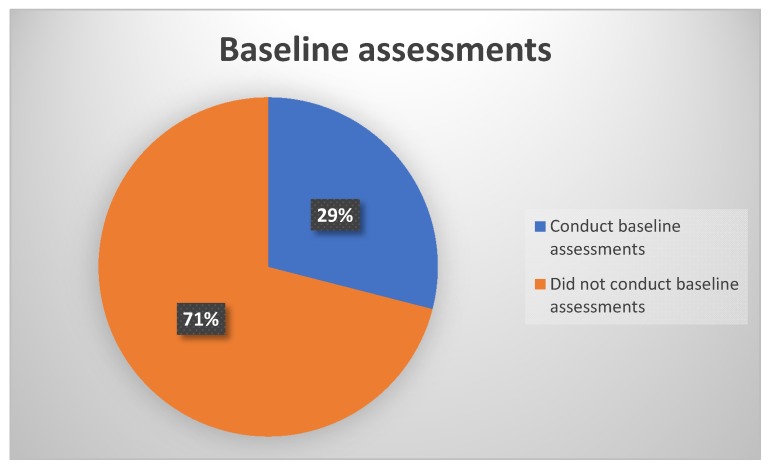
Audiologists’ performance of baseline assessments per patients seen.

In looking at the practice of performing baseline assessments prior to treatment initiation or within 24 hours post treatment initiation, results, as depicted in Figure 2, showed that more than half of the participants 71% (n = 22) did not conduct baseline assessments prior to treatment initiation for all their patients on treatment with ototoxic drugs. Consequently, only a third 29% (n = 9) of the participants reported conducting baseline assessments for 80%−100% of their patients’ assessments. This 29% comprised of audiologists who work in public sector facilities dedicated to the treatment of TB, where their main job is to conduct ototoxicity assessments and management.

The findings of the majority of participants not conducting baseline measures is contrary to international standards, such as those by the AAA [18], as well as HPCSA [4] guidelines. These guidelines suggest that all patients should receive baseline assessments prior to treatment with ototoxic medication. The implications of this omission are significant, as without a baseline assessment there are no hearing thresholds available to compare performance to, to determine the changes in hearing sensitivity, and to implement preventive measures should an ototoxic hearing loss occur. Furthermore, lack of baseline measures prevents early detection and identification, as well as early intervention of ototoxicity. Ultimately, this gap in practice limits opportunities for the adjustment of medication dose and/or frequency, the implementation of early rehabilitative strategies for enhanced patient’s quality of life, as well as patient counselling about possible otologic side effects. Patient counselling enhances treatment adherence [8,11,19], which is key to treatment success.

### 3.3. Frequency of Assessments

As far as the frequency of ototoxicity monitoring was concerned, no participants reported conducting the recommended bi-weekly monitoring to all patients requiring it. A majority 71% (n = 22) of the sample indicated conducting bi-weekly audiological monitoring on less than 10% of their patients requiring ototoxicity monitoring, while the rest of the sample 29% (n = 9) reported that more than 50%, of their patients receive bi-weekly audiological monitoring. These are the same participants who reported conducting baseline assessments.

These findings are concerning as they continue to reveal a significant weakness in the translation of policies/guidelines into practice. Such practice limits early identification of ototoxic changes and impedes medical intervention to conserve hearing function in this population. Govender and Paken [3] found similar findings in their study in the same context. These authors found that most audiologists do not conduct bi-weekly audiological monitoring due to lack of resources or patients that only arrive for audiological monitoring once the hearing impairment is severe. In another study, Harris, Peer and Fagan [8], found that there is huge paucity in audiological monitoring in ototoxicity and this is due to lack of knowledge of guidelines, financial pressure and budgetary demands from life-threatening and/or communicable diseases. Without regular audiological monitoring, hearing threshold shifts will be more difficult to detect thus leading to delayed intervention. This will lead to increased burden of disease and impact on the patients’ quality of life, which are all preventable with adherence to evidence-based practice [20].

### 3.4. Types of Assessment Measures Used

As far as the types of assessment measures used to assess patients with a high-risk for ototoxicity were concerned, the findings, as depicted in Table 1, indicate that there is generally no standard consistent battery of assessments that is used. The results indicate that 70% (n = 21) of the participants use ultrahigh frequency pure tone audiometry, with DPOAEs included by approximately 30% (n = 10) of the participants. Otoscopy and immittance audiometry are the only measures that were found to be the most widely and consistently used with close to 100% (n = 30) of the participants reporting using them.

Findings with regards to equipment used also revealed that the type of equipment used for each patient could change over the period of monitoring, so inconsistent comparative data were available rendering the ototoxicity monitoring programmes insufficient and inefficient. Current findings raise important implications for resource procurement for clinics where ototoxicity is part of the caseload seen. Furthermore, they raise implications about ensuring consistent use of reliable and valid measures for repeated measures to ensure ability to closely compare and contrast the results as part of the monitoring protocol. The high, prevalent use of otoscopy and immittance audiometry on their own in this population is of concern since outer and middle ear function are not the main concern in general ototoxicity monitoring, unless these measures are coupled with additional measures. Immittance is an important measure when used within a test battery approach that includes DPOAEs, where conductive hearing loss may be present, and its presence affects the sensitivity and validity of otoacoustic emissions.

### 3.5. Criteria Used

According to the HPCSA [4] guidelines, audiologists should have criteria to determine changes in patients’ hearing due to ototoxic medication, and this should be decided on before the patient receives the medication. The HPCSA criteria state the following: ≥ 20 dB pure tone threshold shift at a single frequency, ≥ 10 dB shift at two consecutive frequencies or threshold response shifting to “no response” at three consecutive frequencies. Once the presence of an ototoxic shift has been identified; the adverse effect on hearing ability must be graded in accordance with an adverse event scale, specific to hearing. In the HPCSA guidelines, this includes the use of four grades—where grade 1 represents a threshold shift or loss of 15–25dB relative to baseline, averaged at two or more contiguous frequencies in at least one ear, and grade 4 depicts profound bilateral hearing loss >90dBHL. In this study, 77% (n = 24) of the sample reported using the grading system suggested by the HPCSA, but it is important to note that 23% (n = 7) of the sample did not use any system to grade the changes in hearing sensitivity. In terms of the HPCSA grading criteria used to grade the severity, 11% (n = 6) of the participants reported not using this grading criteria and approximately 89% (n = 25) use the suggested criteria to grade the severity of the hearing impairment once a shift had been identified. The use of a grading system and severity criteria, singly or in combination, where other aspects of the ototoxicity monitoring protocol are not in place has minimal value or benefit in this context. Specifically, where a) baseline measures have not been obtained, or b) baseline measures have been obtained, but inappropriate assessment measures were used, and c) frequency of monitoring was not regular. These limitations in an ototoxicity monitoring protocol impact analysis of data, therefore preventing the appropriate use of a grading system for decision making. The lack of evidence-based practice has cascading effects for the ototoxicity monitoring and management programme.

### 3.6. Ototoxicity Management

With regards to the current management practices of patients with ototoxicity, results indicated that the majority of participants (65%) reported providing ototoxicity management within their own institutions. This management involves a combination of various options for 35% of the participants, specifically: a) inform a doctor to explore strategies for medical intervention, b) patient is given hearing aids or other amplification devices, c) patient is enrolled into an aural rehabilitation intervention, d) asking the nurse to monitor ototoxicity signs such as tinnitus as well as provide referrals, as well as e) informing the pharmacist to make recommendations for oto-protective treatments and less ototoxic medications. The rest of the sample reported the provision of hearing amplification (n = 8), aural rehabilitation (n = 6), monitoring patients’ hearing for the subsequent 3−6 months post-ototoxic medications treatment (n = 2) and the referral of the patients for diagnostic audiological assessment (n = 4). These management strategies are in line with international guidelines as well as HPCSA guidelines. Of the participants that reported making referrals for attending physicians/practitioners, almost all reported not receiving any positive collaboration from the practitioners concerned. This is consistent with Wium and Gerber [21], who found that other health practitioners (i.e., doctors, nurses, pharmacists) are unaware of ototoxicity and the effects thereof, and this explains their reluctance to collaborate with audiologists. This raises implications for audiologists, who should increase awareness about ototoxicity amongst these professionals, and should strengthen their patients’ advocacy role for collaborative and efficacious management of patients on ototoxic medications.

### 3.7. Collaborative Work

In terms of whether the multidisciplinary approach is effective for ototoxicity assessment and management within the South African context, the results shown in Table 2 reveal that there are a number of barriers with the effective implementation of this approach. Approximately 100% of the participants reported that the approach is vital in ototoxicity assessment and management practices, however, its efficiency is negatively impacted by a number of factors. In terms of effectiveness of the approach, 31% (n = 7) of the participants reported that the approach is effective only if team members are dedicated and work well together. Majority of the participants 69% (n = 24) felt that the approach was not effective because of various factors including a) lack of trust amongst professionals, b) the late referrals to audiologists, where patients are only referred once they have a severe hearing loss, as well as c) the large caseloads and the hectic schedules of clinicians. Moreover, participants reported that team members’ lack of knowledge regarding ototoxicity, communication amongst the team members as well as training in ototoxicity are some of the challenges that hinder appropriate ototoxicity management within a multidisciplinary team.

Lack of collaborative working relationships between the multidisciplines involved in management of patients on ototoxic medications is not a new finding. Khoza-Shangase and Jina [22] found that although South African general practitioners appeared to be aware of ototoxicity and its symptoms, as well as the audiological services available to them, they did not make use of these audiological services as part of their patient care. In that study, it was concluded that general practitioners focus and place high priority on the patient’s diagnosis over an invisible side effect such as ototoxicity [22]. In another study, Wium and Gerber [21] found that practitioners do not make the referrals to audiologists due to time constraints, as well as due to insufficient knowledge from the practitioner and the patient about ototoxicity signs and symptoms. Internationally, current findings echo those from New Zealand where there is no nationally accepted ototoxicity monitoring programme practice pattern such that the state of monitoring is reported to be “poorly understood” [23]. In a study by Steffens et al. [24] in New Zealand, findings revealed a need for better collaboration between disciplines involved; with oncologists and audiologists being on opposing sides. For example, oncologists reported that information provided by audiologists guided oncology treatment decisions when possible, whilst audiologists suspected that their information was either not used or did not influence treatment decisions. In the current study, results seem to indicate that the multidisciplinary approach is theoretically seen as relevant and important, but its implementation almost impractical within the South African context. This situation might be changed by the impending National Health Insurance (NHI) implementation which has a health teams approach within a re-engineered primary health care model. This approach has preventive care, where ototoxicity prevention is located, as a priority [25].

### 3.8. Additional Analysis

In an attempt to establish if there were any trends in the data that could explain the outcomes in terms of current practice, factors such as level of qualification, place of employment, sector of employment, and length of employment were carefully qualitatively scrutinized. Of these factors potentially influencing adherence to guidelines, institution of employment, specifically employment in TB hospitals, seemed to have a positive influence on current practice, possibly due to the focused nature of the audiologists’ scope of practice in these hospitals as well as the availability of resources—as ototoxicity monitoring forms part of standard hospital care. This is a single big difference in these specialized hospitals, where resources are focused and dedicated. The level of education of the participants appeared to have no influence in current findings. This was not expected as ototoxicity assessment and management forms part of the HPCSA minimum standards for undergraduate qualification in Audiology in South Africa, with postgraduate training comprising of research only, research report and research coursework, with no clinical training at postgraduate level.

## 4. Conclusions

Up until recently there has been limited research that investigates the current ototoxicity assessment and management practices in South Africa. This is a serious gap in evidence on ototoxicity monitoring, which Campbell and Le Prell [26] emphasize should cover detection and monitoring as well as grading of adverse events in any context. This is an important area for research because of the increased risk of communicable diseases that require ototoxic medication for treatment. The lack of clinical guidelines that have been developed for the South African context, until recently, has exacerbated this situation. Current findings indicating training in ototoxicity assessment and management for all audiologists has not translated well to the practices exhibited by the same audiologists within this South African context are concerning. Similar findings have been found in early hearing detection and intervention (EHDI) studies within the South African context, where implementation of knowledge has been found to be impeded by contextual challenges. In this study; specifically, practices around performance of baseline measures as well as frequency of assessments during monitoring were found to be less than optimal, and did not adhere to guidelines. Limited consistency in the types of assessment measures used in the assessment batteries was found, with inconsistent application of standardized criteria used to establish ototoxicity and the grading approaches adopted. Ototoxicity management is influenced by a number of barriers including poor collaborative work between the disciplines involved in this population.

This highlights factors such as lack of translating knowledge, policies and guidelines into practice, as well as a clinical environment that is not conducive to this translation. This is a consistent finding in most studies around policies, guidelines and regulations within the South African context, where what is on paper does not get translated into practice for various reasons; key to which are resource constraints. Current findings seem to suggest that sufficient graduate training and the existence of policies and guidelines do not guarantee implementation, since this could be impacted by a number of factors, including availability of equipment, workload, training of team members in ototoxicity, and so on. The lack of standardized valid and reliable test battery and sensitive assessment measures nationally, with poor regular monitoring and follow up of patients on ototoxic medications limits chances of early identification followed by intervention in this population. The limited collaborative work between audiologists and attending physicians (and the rest of the medical team involved in the management of these patients) to enable prevention of ototoxicity further negatively influences hearing conservation efforts for this already vulnerable population group. This prevents the execution of what Lord [27] refers to as another key purpose of ototoxicity monitoring, over and above provision of feedback to the attending physician about the effects the treatment is having on the auditory system; that of setting expectations for the patient and his/her family about the communication issues that may result from the drug therapy.

Current findings provide some contextually relevant evidence that will contribute toward ototoxicity programmes’ strategic planning, implementation and monitoring. These findings are novel for the South African context as far as implementation of ototoxicity monitoring and management guidelines, on the heels of the 2018 release of the HPCSA guidelines [4]. Furthermore, previous studies have had physicians as participants, have looked at practice in specific conditions such as cancer or TB, while this study focused on audiologists with general ototoxicity assessment and management perspective [21,22,23,24]. Nonetheless, these findings should take cognizance of the identified methodological weaknesses of the study. The small sample size of this study, although a finding on its own in terms of practicing audiologists’ interest in ototoxicity assessment and management, particularly in the private healthcare sector in South Africa, must be taken into consideration when interpreting current findings. Furthermore, the fact that reasons for why certain practices were not followed were not explored in the current study is an identified limitation and raises implications for future studies. These findings have significance for the clinical training (education) of all team members, continued professional development, policy formulation by healthcare policy makers, resource planning and allocation (including both equipment and relevant staff), as well as awareness campaigns around collaborative work within the South African context. All these implications have the aim of ensuring that current practice improves in order for the guidelines to be effectively and efficiently implemented. This is important as these guidelines have carefully taken the South African context into consideration without compromising the goal of ototoxicity monitoring programs. These implications also have a goal of ensuring that barriers identified in this study are addressed. Because the current study was an initial assessment of current practice in light of the HPCSA guidelines, future studies should collect data that can establish causal links between some of the barriers identified, e.g., lack of staff or equipment, and the quality of ototoxicity assessment and management practices.

## Figures and Tables

**Table 1:** Equipment used to perform audiological assessment for patients at risk for ototoxicity.

Equipment Used for Assessments:	Participants Number (%):
Otoscopy and Immittance Audiometry	30 participants (97%)
Ultra-high frequency pure tone audiometry	21 participants (70%)
DPOAEs	10 participants (30%)

**Table 2:** The effectiveness of the multidisciplinary approach in South Africa.

The Effectiveness of the Multidisciplinary Approach in Ototoxicity	Percentages:
Approach is effective for this context:	23% (n = 7)
Approach is not effective for this context due to:	69% (n = 24)
Lack of trust amongst professionals	19% (n = 6)
Referrals to audiologists once a hearing loss has developed	6% (n = 2)
Large caseloads	13% (n = 4)
Hectic schedules of clinicians	10% (n = 3)
Lack of knowledge in ototoxicity	13% (n = 4)
Poor communication amongst clinicians	16% (n = 5)

## Appendix A

**Survey:** What are the current Ototoxicity assessment and management practices in South Africa?

Please take a few minutes to fill out this survey on the current practices of South African audiologists for the assessment and management of ototoxicity in adults. All answers from the survey will be kept confidential and used solely for academic purposes. Thank you for your participation.

Informed consent: By completing this survey, I am giving consent for the information that I have supplied to be used by the researcher (please tick box as appropriate).

**Demographics:**	
**1. Please state your gender**	
☐ Male ☐ Female ☐ Other	

**2. What is your highest level of education?**	
☐ Master’s degree	☐ Clinical doctorate degree (e.g., Au.D)
☐ Undergraduate degree	☐ Research doctorate degree (e.g., Ph.D)
Other (Please specify: ____________________)	

**3. In what type of setting do you practice?**	
☐ Private	☐ Public
**OTOTOXICITY ASSESSMENT & MANAGEMENT PRACTICES:**	

**4. Are you involved in ototoxicity assessment and management?**	
☐ Yes ☐ No	
**5. On average, how many patients receive baseline assessment prior to treatment initiation or within 24 hours post treatment initiation with ototoxicity medication?** (*Please indicate a numeric value*) ____________________	
**6. On average, how many patients receive bi-weekly audiological monitoring?** (*please indicate a numeric value*) _____________________	
**7. What type of assessment is used to form a baseline audiogram for high-risk patients?** (*Please tick all the appropriate answer/s*) ☐ Case History ☐ Otoscopy ☐ Immittance testing ☐ Pure Tones (basic standard frequencies) ☐ Pure Tones (including ultrahigh frequencies) ☐ Bilateral DPOAEs ☐ ABR ☐ Other (please specify ____________________________________)	
**8. What bi-weekly audiological monitoring components are included?** ☐ Bilateral otoscopic examination ☐ Bilateral pure tone air conduction testing (basic standard frequencies) ☐ Bilateral pure tone air conduction testing (including ultrahigh frequencies) ☐ Bilateral DPOAEs ☐ ABR	
**9. What criteria do you use to determine an ototoxicity shift in hearing due to ototoxicity?** ☐ ≥ 20dB pure tone threshold shift at a single frequency ☐ ≥ 10dB shift at 2 consecutive frequencies or threshold responses ☐ A no response at 3 consecutive frequencies ☐ Other (please specify ____________________________________)	
**10. What criteria do you use to grade the severity of the ototoxicity shift?** ☐ Grade 1: threshold shift or loss of 15- 25dB relative to baseline ☐ Grade 2: threshold shift or loss at >25-90dB ☐ Grade 3: hearing loss indicates hearing intervention ☐ Grade 4: Indication for cochlear implants ☐ Other (please specify): ____________________________________	

**11. What management options are available to offer to patients identified with a shift:** (*please tick all the appropriate areas*) ☐ Inform doctor to explore strategies for medical intervention ☐ Nursing staff to monitor ototoxicity signs and give referrals ☐ Pharmacist to make recommendations for otoprotective treatments such as well as less ototoxic medication ☐ Patient is given hearing aids or other amplification devices ☐ Patient is enrolled into an aural rehabilitation intervention including appropriate clinician ☐ Patient’s hearing is monitored up 3–6 months after termination of medication. ☐ Other (*please explain*): ____________________________________________________________ ____________________________________________________________ ____________________________________________________________	

Thank you for your participation.
